# Evidence-Based Prediction of COVID-19 Severity in Hospitalized Children

**DOI:** 10.1155/2022/1918177

**Published:** 2022-04-09

**Authors:** Shahnaz Armin, Mohammadreza Mirkarimi, Zahra Pourmoghaddas, Marjan Tariverdi, Ahmad Shamsizadeh, Mohsen Alisamir, Maryam Mohammadian, Mohammad Bagher Rahmati, Sedigheh Rafiei Tabatabaei, Roxana Mansour Ghanaiee, Seyed Alireza Fahimzad, Ayeh Yaraghi, Seyedeh Mahsan Hoseini-Alfatemi, Noushin Marhamati, Farzad Esmaeili Tarki, Armin Shirvani, Abdollah Karimi

**Affiliations:** ^1^Pediatric Infections Research Center, Research Institute for Children's Health, Shahid Beheshti University of Medical Sciences, Tehran, Iran; ^2^Aboozar Children's Medical Center, Ahvaz Jundishapur University of Medical Sciences, Ahvaz, Iran; ^3^Pediatrics Infectious Disease Department, Isfahan University of Medical Sciences, Isfahan, Iran; ^4^Department of Pediatric, Clinical Research Development Center of Children Hospital, Hormozgan University of Medical Sciences, Bandar Abbas, Iran; ^5^Infectious and Tropical Diseases Research Center, Health Research Institute, Ahvaz Jundishapur University of Medical Sciences, Ahvaz, Iran; ^6^Department of Pediatric Infectious Diseases, Children's Clinical Research Development Center, Hormozgan University of Medical Sciences, Bandar Abbas, Iran; ^7^Virtual School of Medical Education and Management, Shahid Beheshti University of Medical Sciences, Tehran, Iran

## Abstract

**Objective:**

In this study, by using clinical and paraclinical characteristics, we have aimed to predict the severity of the disease in hospitalized COVID-19 children.

**Method:**

This cross-sectional study was conducted on medical records about epidemiologic data, underlying diseases, symptoms, and laboratory tests from March to October, 2020, on 238 hospitalized confirmed COVID-19 paediatric cases in several children's hospitals of Tehran, Ahwaz, Isfahan, and Bandar Abbas.

**Results:**

From 238 patients, 140 (59%) were male and most of them were in the age group of 1 to 5 years (34.6%). Among all hospitalized patients, 38% had an underlying disease and in total, 5% of cases were expired.

**Conclusion:**

Determining patient severity is essential for appropriate clinical decision making; our results showed that in hospitalized pediatric patients, by using several variables such as SGOT, CRP, ALC, LDH, WBC, O_2_sat, and ferritin, we can use clinical and paraclinical characteristics for predicting the severity of COVID-19.

## 1. Introduction

The novel severe acute respiratory syndrome-coronavirus-2 (SARS-CoV-2) infection has appeared in the city of Wuhan, China, in December 2019, and then spread to other countries all around the globe, including Iran [1, 2]. Since COVID-19 is transmitted by droplets, daily activities can diffuse contaminated droplets by close contact. The children, forming an important part of every society, have been the target of this virus as well as all of the other age groups [3].

Early symptoms of COVID-19, such as fever, sore throat, cough, illness, rhinorrhea, diarrhea, vomiting, and stomach pain, are analogous between children and adults. Although the majority of children infected by COVID-19 are asymptomatic, silent carriers, or have mild constitutional symptoms, it has been shown that underlying chronic disease can lead to severe infection in children [4, 5].

Based on studies, the frequency of severe and fatal COVID-19 infection in children is very low [6, 7] and it may be attributed to as follows:

  Being less present in communities [6, 8, 9]. Most children were infected by family members.
  Less exposure to air pollution and cigarette smoke.
  Lower maturity levels of angiotensin-converting enzyme 2 (ACE2) receptors, which are the binding targets of SARS-CoV-2 [8].
  More effective inherent immune response and better condition of respiratory tracts. [10, 11].

According to the Center for Disease Control and Prevention (CDC), 2% of infected COVID-19 cases in China were children [12], and this index was about 6.4% in South Korea [13]. The mean age of the confirmed COVID-19 children cases was 6.7 years [14].

Since a comprehensive study in predicting the severity of hospitalized confirmed COVID-19 paediatric cases in the world based on clinical and paraclinical variables has not been performed, we have designed this study to find simple patterns for the prediction of severity in hospitalized children.

## 2. Method

This multicentral cross-sectional study was carried out from March 2020 to October 2020, on 238 hospitalized confirmed COVID-19 paediatric cases in several major educational paediatric hospitals located in four provinces of Iran, including Tehran, Ahwaz, Isfahan, and Bandar Abbas.

All of the hospitalized children who had a confirmed diagnosis of COVID-19 and were under 18 years old participated in this study. Confirmed COVID-19 infection was defined as one positive real-time reverse transcription-polymerase chain reaction (RT-PCR) test on the nasopharyngeal sample.

Medical records were reviewed and patient information about epidemiologic data, past medical history, underlying diseases, history of participating in social gatherings, familial exposure history, symptoms, paraclinical data (laboratory results, radiologic findings, etc.), and treatment information were all collected in standardized forms.

In this study, for prediction of severity, we exerted the laboratory tests that were performed routinely in the first days of admission. The unfavorable events evaluated as signs of severity during hospitalization are as follows: the need for oxygenation with reservoir, mechanical ventilation, taking a vasoactive drug, IVIG, corticosteroid therapy, and death.

### 2.1. Statistical Analysis

The collected data were analyzed by using the IBM SPSS and STATA 16 software, version 25.

The backward stepwise logistic regression method was used to prognose the severity of the disease, and variables such as underlying disease, tachypnoea, fever, age, early O_2_sat, laboratory tests, chest CT scan, and radiography findings were included as potential predictors.

To predict the length of stay (LOS) in hospitals based on clinical and paraclinical information, the stepwise regression method based on a generalized linear model, with gamma distribution was used, and the correlation coefficient was calculated with 95% confidence interval (CI). *P* values of less than 0.05 were considered statistically significant.

## 3. Results

Medical records of 238 COVID-19 hospitalized pediatrics were evaluated, 98 (41%) patients were female and 140 (59%) patients were male. Of these, 15.6% were <1 year old, (34.6%) were 1–5, (23.6%) 5–10, and (26.2%) above years old.

38% of all patients had at least one underlying disease. 41% of patients had a history of contact with suspected or confirmed cases and 11% had participated in gatherings. Among these hospitalized patients, 59% had pulmonary involvements and 12 cases (5%) were expired.

In our study, the use of antibiotics and antivirals in hospitalized children with a diagnosis of COVID-19 is 82% and 39%, respectively. Also, the most prevalent clinical symptoms were fever (73%), cough (39%), vomiting (29%), diarrhea (18%), and myalgia (13%).

Overall, our results have demonstrated that we can estimate the extent of severity by some existing clinical and paraclinical variables in hospitalized pediatrics.

### 3.1. Predicting the Severity of COVID-19 by Using Paraclinical Variables

**Figure 1:**
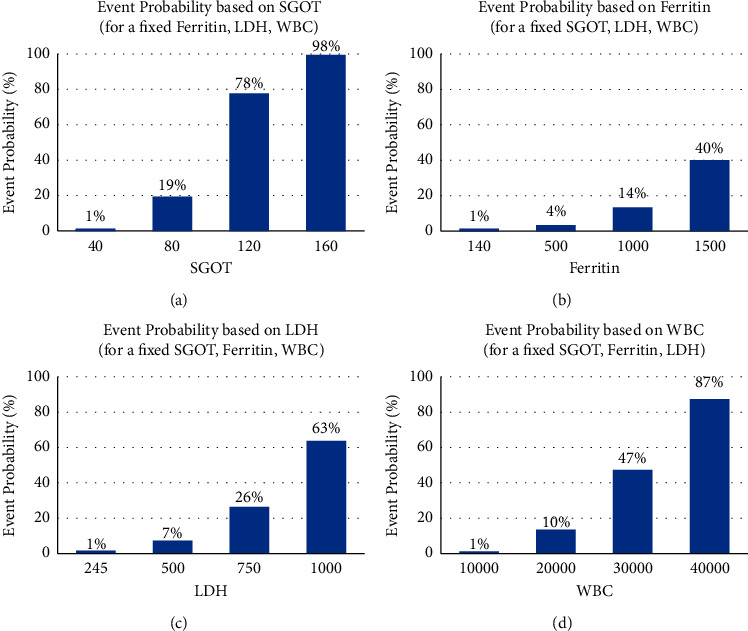
(a, b, c, d) Predicting the probability of event as a sign of severity by considering ferritin, WBC, LDH, and SGOT. (a) Event probability based on SGOT. (b) Event probability based on ferritin. (c) Event probability based on LDH. (d) Event probability based on WBC.

  (1) By considering ferritin, WBC, LDH, and SGOT, we predict the probability of the event as a sign of severity. When all of these elements are in the normal range, the probability of unfavorable event will be around just 1%. The increase in any of the valuables will elevate the probability of the unfavorable events. Also, when the level of each factor increased twice with hyperferritinemia, a synergistic effect was observed, resulting in the probability of the unfavorable event reaching 96%.
  We have attempted to form figures associated with every four factors.  Figure 1(a) shows how SGOT affects the event probability for a fixed ferritin of 140, LDH of 245, and WBC of 10000.  Figure 1(b) demonstrates how ferritin impacts the event probability for a fixed SGOT of 40, LDH of 245, and WBC of 10000.  Figure 1(c) portrays how LDH affects the event probability for a fixed SGOT of 40, ferritin of 140, and WBC of 10000.  Figure 1(d) demonstrates how WBC impacts the event probability for a fixed SGOT of 40, ferritin of 140, LDH of 245. Based on a one-by-one comparison of these four figures, it could be clearly understood that the rise of SGOT compared to other factors, has a steeper effect on the event probability scoring.
  (2) By using CRP, ALC, SGOT, the probability of events was estimated. We discovered that while all variables are in the normal range, the probability of events is 1%, and when these three variables increased, the effect of synergism was almost noted. The effects of each variable are shown in Table 1.
  (3) We have presented the third based on having/not having an underlying disease. We found out that underlying disease as a risk factor had a significant relation with SGOT and CRP variables in the prediction of the event. If CRP and SGOT are in the normal range, the probability for severity in children with underlying diseases will be 18% (*P* value = 0.018), while the rate will be 1% in children without any underlying disease.
  The probability of events considering SGOT and CRP in patients with/without underlying disease are shown in Table 2, and we could see how impactful solely SGOT was in cases without underlying disease.
  (4) Based on the initial laboratory data, we attempted to predict the LOS, an important factor for both the family and the physician. As it is demonstrated in Table 3, the increase in the amount of platelet to 500000 could be solely an important predictive factor but all of the four valuables have a synergistic effect and when an abnormality occurs in any of them, the LOS increases to 15 days.
  (5) The decrease in O_2_ saturation is a common sign in hospitalized COVID-19 patients, and in our study, the association of O_2_ saturation with other variables was investigated. The relation of the CRP level and O_2_ saturation with severity was seen; oxygen saturation drops to 90% and CRP rises, and the probability of unfavorable event increases to 57%.

## 4. Predicting the Severity of COVID-19 by Using Clinical Variables

  (1) We found that in patients with neurological symptoms such as headache, convulsion, or loss of consciousness as the common presentations (12.5%) in our cases, ALC and CRP can help to predict events.
  (2) As the last results, the severity of the disease can be predicted based on the presence of tachypnea and the level of ferritin; if the patient has tachypnea and the ferritin's level is 140 *μ*g/L (normal range), the chance of an event is 50%, and when it increases to 500 *μ*g/L, this probability of event rises to up to 70%.

## 5. Discussion

COVID-19 has been a quickly evolving pandemic that has been putting a strain on healthcare systems. Symptoms range from moderate fever to ARDS, which complicates diagnosis, prognosis, and further supervision. It is, therefore, crucial to determine the status of the patient on time.

In this study, the severity of COVID-19 in hospitalized cases in COVID-19 wards is predictable based on clinical and paraclinical evidence. Overall, the key goal of severity prediction is to help medical decision making. It is therefore important to define a target demographic for which predictions serve a therapeutic purpose, and a representative data set for which a prediction model could be built and proved to be valid. On the contrary, in our research, the study population was quite defined; according to our analysis, this is one of the first studies to predict the severity of COVID-19 in children, which is defined by the percentage of probability.

As a first step, the definition of severity should be described. In the study conducted by Karimi et al., the scoring model helps clinicians to detect and manage COVID-19 patients [15]. Zachariah et al. [16] defined severity as the need for ventilatory or hemodynamic support, and Dong et al. defined it as having respiratory symptoms, dyspnea, and hypoxemia [10]. Our study has included a set of events that present the severity of COVID-19 disease: using a ventilator, oxygen with reservoir, taking vasoactive drug, IVIG or corticosteroid therapy, and death.

In our study, the highest number of hospitalized COVID-19 paediatric cases is in the age group of 1–5 years, whereas in reports from China and Italy, this rate among children was those aged <1 year and under 6 months, respectively (105–6,29,30). Based on CDC, the highest hospitalization rate was among children aged <2 years (24.8%) [17].

Considering that in previous studies, the prediction of COVID-19 severity was mentioned in adults [18, 19], and obviously, since we cannot generalize those results to all age groups, therefore, we decided to design several severity prediction patterns based on clinical and paraclinical symptoms in children. In the first, based on four variables when SGOT is 2 and 4 times higher, there is a 19% and 98% chance of severity of disease, respectively. In fact, due to its nonspecificity and different sources of secretion, we consider it as a sign of extensive pathological effects of SARS-CoV-2 and inflammation of multiorgan systems. Furthermore, simultaneous examination of AST and ferritin in other studies found that these two items increased the risk of disease severity [20].

In this study, we delineated that, if the ferritin level increases significantly while the rest of the variables stay normal, there is a 40% probability of an event, which in turn could reflect the nonspecificity of ferritin and its role in inflammation. On the other hand, when the level of LDH and WBC was higher than the normal range, the probability of an unfavorable event elevated, indicating the correlation between inflammation and this disease. Similarly, other studies have mentioned that an increase in LDH as an inflammatory marker accompanies the severity of disease and tissue damage [20, 21].

Along with our results, in COVID-19 patients, it has been proven that the release of proinflammatory cytokines, resulting from an elevated CRP, LDH, and ferritin concentration seems to be responsible for disease progression [22–24].

Since, in many centers, a ferritin test could not be performed or it is performed with a delay, we designed the second severity prediction without ferritin to ease the decision-making for the doctors who don't have access to the ferritin test. The increase in CRP and SGOT and the decrease in ALC had a synergistic effect on the probability of the event. In line with our study, increased levels of CRP and liver enzymes in other studies could also be deemed as an indicator of disease severity [25–30].

Because in other studies, the underlying disease is viewed as an important factor in the severity of the disease and is known to be an important indicator for the hospitalized [31], we decided to also check the presence or absence of underlying disease with the possibility of an event and severity of the disease. The information obtained from this section can be efficacious for clinicians to triage, prescribe personalized treatments and monitor clinical progress. Moreover, it can help the proper allocation of resources as well as reduce mortality and morbidity [32].

These data showed with normal SGOT and CRP the probability of an event will be 1% and 18% in cases without and with underlying disease, respectively. This depicts that the presence of at least one risk factor can be of importance in the severity of the disease. Besides, the 3-fold increase in SGOT levels in the presence of a normal CRP elevates the probability of an event up to 86% in people without underlying disease and 99% in people with underlying disease. This very fact again portrays the significance of SGOT as an inflammatory factor for the prediction of severity.

Accordingly, another study conducted on adults showed that the presence of an underlying disorder and the level of CRP could be valuable markers for progression from nonsevere to severe cases [33].

The COVID-19 paediatric patients had a shorter length of stay compared to adults [34]. This is an important matter for the patient's family and the physician, especially in places where the number of beds is limited. However, we can estimate the duration of hospitalization in children based on the factors we are dealing with. In a study conducted in the west of Iran, lymphopenia, LDH, CRP, and WBC with clinical indicators including tachypnea and hypoxia were taken into consideration as predictors for the length of hospital stay, and the need for important treatment measures as well as the severity of COVID-19 infection [35].

Based on our results, when ALC is less than 1000 and Plt, WBC, and CRP, respectively, increase to 500000, 15000, and 20, the hospitalization time will expand to 15 days, but if the abovementioned factors are in the normal range, the hospitalization period could be is reduced to 5 days.

In a study carried out by Kermali et al. in 2020, the level of lymphocytes and platelets was shown to be significantly lower in severe patients as against nonsevere patients [36].

In the presence of neurological symptoms (12.5%) associated with decreased ALC and CRP, tachypnea with an increased level of ferritin can demonstrate the severity of COVID-19 infection in hospitalized children.

The data published by Columbia University Irving Medical Center have reported that 34% of hospitalized children had neurological symptoms, which is significantly higher than the results in our study [37]. This discrepancy could be explained by the different sample sizes and the different number of symptoms that were considered, according to the information received from patients to define neurological symptoms.

One should bear in mind that COVID-19 prediction models as a whole might be at great risks of bias, primarily due to several reasons. Firstly, because the selection process of control cases is usually far from representative. Secondly, the patients who have not encountered an event by the end of the study get excluded from the analysis. Therefore, further studies might be needed to shed more light on these concerns and check the reliability and efficiency of these prediction patterns.

As for the limitations in this study, we should point out that because of the large number of missing data for some laboratory markers (for example, D-dimer, BUN, PT, etc), these variables were excluded to avoid noise predictors. Due to the limited cases, we did not specify the type of underlying disease.

## 6. Conclusion

Evaluation of SGOT, CRP, ALC, LDH, WBC, O_2_sat, and ferritin levels in children with COVID-19 can be an indication for hospitalization and a predictor of disease severity, aiding clinicians in the management of patients. The high costs of long hospitalizations, as a burden on the government and families, underline the importance of the length of stay in hospitals. Needless to mention that longer hospitalizations put the carers who stay within the hospital at greater risk of nosocomial infections.

## Figures and Tables

**Table 1:** Effect of SGOT, ALC, and CRP in predicting the probability of event without considering ferritin.

ALC	SGOT	CRP	Event (%)
3000 3000 3000 3000	40 40 40 40 40 40 40	10	1
		20	1
		40	2
		70	3
1000		10 10 10 10 10	3
500			4
100			4
3000	80		5
3000	120		19
500	80	40	22
500	120	40	56

**Table 2:** The probability of event in children with/without underlying disease.

SGOT	CRP	Probability event (without underlying disease) (%)	Probability event (with underlying disease) (%)
40	10	1	18
	20	1	25
	40	4	43
	70	10	73
80	10	19	85
120	10	86	99

**Table 3:** Prediction of LOS based on laboratory results

WBC (*P* value = 0.008)	Plt (*P* value = 0.003)	CRP (*P* value = 0.052)	ALC (*P* value = 0.008)	LOS prediction
10000	200000	10	3000	5 days
	200000 200000 200000 200000	10 10	1000	7.5 days
			500	8 days
		40	3000 3000 3000 3000 3000	6.5 days
		70		8.3 days
	500000	10		11 days
15000	200000	10 10		7 days
20000	200000			9 days
15000	500000	20	1000	15 days

## Data Availability

The data used to support the findings of this study are available from the corresponding author upon request.
